# The Effect of Thymoquinone on the TNF-α/OTULIN/NF-κB Axis Against Cisplatin-İnduced Testicular Tissue Damage

**DOI:** 10.1007/s43032-024-01567-y

**Published:** 2024-04-24

**Authors:** Tuba Yalçın, Sercan Kaya, Akın Yiğin, Can Ali Ağca, Deniz Özdemir, Tuncay Kuloğlu, Murat Boydak

**Affiliations:** 1 Vocational Higher School of Healthcare Studies, Batman University, Main Campus, Health Services Vocational School, Room 217, Kültür Neighborhood, Batman, Turkey; 2 Department of Geneticy, Faculty of Veterinary Medicine, Harran University, Şanlıurfa, Turkey; 3 Department of Molecular Biology and Genetics, Bingol University, Bingol, Turkey; 4 Department of Histology and Embryology, Faculty of Medicine, Firat University, Elazig, Turkey; 5 Department of Histology and Embryology, Faculty of Veterinary Medicine, Selçuk University, Konya, Turkey

**Keywords:** Nuclear factor kappa B, OTULIN, Cisplatin, Testicular damage, Thymoquinone, Tumour necrosis factor-alpha

## Abstract

One of the adverse effects of the antineoplastic drug cisplatin (CS) is damage to testicular tissue. This study aimed to examine the potential therapeutic effect of thymoquinone (TQ), a strong antioxidant, against testicular damage caused by CS. In the experiment, 28 rats were used, and the rats were randomly divided into four groups: control (*n* = 7), CS (*n* = 7), CS + TQ (*n* = 7), and TQ (*n* = 7). The experiment was called off after all treatments were finished on day 15. Blood serum and testicular tissues were utilized for biochemical, histological, immunohistochemical, mRNA expression, and gene protein investigations. The testosterone level decreased and oxidative stress, histopathological damage, dysregulation in mitochondrial dynamics, inflammation and apoptotic cells increased in testicular tissue due to CS administration. TQ supplementation showed anti-inflammatory, antioxidant, and anti-apoptotic effects in response to CS-induced testicular damage. In addition, TQ contributed to the reduction of CS-induced toxic effects by regulating the TNF-α/OTULIN/NF-κB pathway. TQ supplementation may be a potential therapeutic strategy against CS-induced testicular damage by regulating the TNF-α/OTULIN/NF-κB axis, inhibiting inflammation, oxidative stress, and apoptosis.

## Introduction

Cisplatin (CS) is a chemotherapeutic drug used in the treatment of many types of cancer [1]. However, the therapeutic use of CS is limited due to its adverse effects on spermatogenesis and fertility [2]. DNA strands break when CS attaches itself to purine nucleotides in DNA. Damaged DNA, RNA, and proteins then trigger DNA repair pathways, which can result in either apoptotic or non-apoptotic cell death. CS is known to disrupt Leydig cell function and cause severe testicular damage by inducing apoptosis of germ cells [3, 4]. On the other hand, an excessive generation of reactive oxygen species (ROS) is linked to CS-induced reproductive damage. Increased ROS-induced oxidative stress results in spermatogenesis being reduced, DNA damage, and an imbalance in the reproductive organs' oxidant-antioxidant system [5]. Studies have shown that CS triggers spermatogenesis abnormalities, lipid peroxidation, ROS formation, and decreased reproductive hormones [6, 7]. However, there is no specific treatment or supplement yet against CS-induced testicular toxicity. Considering that ROS and oxidative stress are involved in the development of CS-induced testicular damage, antioxidant compounds may be considered as a way to reduce the harmful effects of CS [8].

Plant-derived products, which are a safer alternative to synthetic chemicals, are used effectively in many pathophysiological conditions [9]. One of these, black cumin (*Nigella sativa*), has been proven by studies to have many beneficial effects such as antioxidant, antiapoptotic, and anti-inflammatory [10, 11]. However, the bioactive component of *Nigella sativa* is thymoquinone (TQ). TQ has antioxidant, anti-inflammatory, and anti-apoptotic effects and therapeutic potential [12]. A study reported that TQ has a cytoprotective effect against the negative effects of varicocele on testicular tissue and sperm morphology [13]. Another study reported that TQ exerts a protective effect against hypothyroidism-induced testicular damage through its antioxidant, anti-inflammatory, anti-apoptotic, fertility-enhancing, and endocrine modulator activities [14]. Similarly, many studies have shown the beneficial effects of TQ on fertility and spermatogenesis [15, 16].

In general, oxidative stress and inflammation are the primary causal factors that damage cells. It has been reported that the expression of inflammatory cytokines is induced through Nuclear factor kappa B (NF-κB) signaling in CS-induced testicular injury [17]. However, it has also been reported that NF-κB is activated by pro-inflammatory signaling pathways as a result of tissue damage. Inflammation is tightly regulated, including the reversible exchange of signaling proteins [18]. OTULIN, the only known mammalian deubiquitinating enzyme (DUB) that hydrolyzes linear ubiquitin chains from proteins altered by the linear ubiquitin chain assembly complex (LUBAC), is one of the proteins in charge of this regulation. The only DUB that exclusively eliminates linear ubiquitin chains is OTULIN [19]. OTULIN plays an important role in controlling NF-κB activation and apoptosis in cells [20]. OTULIN negatively correlates with NF-κB activation and cell death [21]. On the other hand, proinflammatory NF-κB signaling, activated when tumor necrosis factor-alpha (TNF-α) binds to TNF receptor 1, can cause cell death [22]. It has also been reported that OTULIN plays an important role in the control of TNF-α signaling [23].

Although oxidative stress and inflammation are assumed to be the underlying mechanisms of CS-induced testicular toxicity, the detailed mechanisms of action are still not fully understood. Additionally, although many supplements have been studied to alleviate CS-induced adverse effects, none are yet in routine use. In this respect, this study focused on the therapeutic potential of TQ against CS-induced testicular damage. The purpose of this research was to examine the possible modulatory impact of TQ on the TNF-α/OTULIN/NF-κB axis to guard against testicular tissue damage caused by CS.

## Material and Method

### Experimental design

The Dicle University Animal Experiments Local Ethics Committee, with approval dated 29/03/2022 and a number of 259527, approved the conduct of this investigation. All applications in the experimental design were performed within the scope of ARRIVE guidelines. Both TQ (Cayman, USA) and CS (Koçak Farma, Turkey) were obtained from for-profit businesses to be employed in the experiment. The 28 rats (male, Sprague–Dawley, 8–10 weeks old, 200 ± 20 g) used in the experiment were housed under optimum conditions (23 ± 2 °C, 12 h light cycle, water and feed on demand). Rats used in the experiment were divided into four groups at random, each including seven rats. The control group (*n* = 7) received no treatment. CS group (*n* = 7) received a single dose of CS intraperitoneally at a dose of 7 mg/kg on the first day of the experiment. CS + TQ group (*n* = 7) received a single intraperitoneal dose of 7 mg/kg CS on the first day of the experiment and 10 mg/kg/day TQ by oral gavage until the end of the experiment. TQ group (*n* = 7) received 10 mg/kg/day TQ by oral gavage until the end of the experiment.

The doses and applications of CS and TQ used in the experiment were determined by previous studies [4, 24, 25]. On the 15th day, intracardiac blood samples were taken from the rats and the experiment was terminated by sacrificing the rats under anesthesia (ketamine-xylazine). At the end of the experiment, the final body weights of the rats were measured. Testicular tissues were quickly taken completely separated from other tissues and washed with cold PBS. Testicular weights were then measured and relative testicular weight was calculated (Relative testis weight = Testis weight (g)/ final body weight × 100) [17]. Blood serum samples and testicular tissues were stored at -80 °C until analyzed (In order to perform Real-Time Polymerase Chain Reaction (PCR), certain testicular tissues were frozen in liquid nitrogen). Some testicular tissue samples taken for Real-Time PCR studies to evaluate gene expression in testicular tissues were also stored at -80 °C until used. In addition, testicular tissues were placed in Bouin's fixative solution for histopathological and immunostaining.

### Biochemical Analyses

Serum testosterone and Gonadotropin-releasing hormone (GnRH) levels were determined by thawing blood serum samples stored at − 80 °C only once at the end of the experiment. An automatic biochemical analyzer (Centaur XPT ADVIA, Siemens) was used to determine serum testosterone levels. Additionally, GnRH levels in serum samples were determined by enzyme-linked immunosorbent assay (ELISA). Testicular tissues were homogenized (+ 4 °C, 10% phosphate buffer solution, 5000 rpm, 20 min) and supernatants were taken. The supernatants were analyzed by ELISA to determine the levels of Malondialdehyde (MDA), Catalase (CAT), OTULIN, and Superoxide dismutase (SOD) in testicular tissue. ELISA kits were used according to the manufacturer's instructions GnRH (Elabscience, USA), (OTULIN (SunRed, China), MDA, CAT, and SOD kits were purchased from Fine Test (China)). GnRH kit sensitivity was 0.19 ng/ml and the test range was 0.31–20 ng/ml. MDA kit sensitivity was 4.688 ng/ml, test range was 7.813–500 ng/ml. CAT kit sensitivity was 18.75 mlU/ml, test range was 31.25–2000 mlU/ml. OTULIN kit sensitivity was 4,617 pg/ml, test range was 5–100 pg/ml. The sensitivity of the SOD kit was 0.469 ng/ml and the test range was 0.781–50 ng/ml.

### Histopathological evaluations

Testicular tissues were fixed in Bouin's solution for 12 h. It was dehydrated in different alcohol series and embedded in paraffin. 5 µm sections taken from the blocks were stained with hematoxylin and eosin (HE) for histopathological examinations. The preparations were photographed and examined under a light microscope (Leica-DM2500/MC170 HD, Germany). Histopathological evaluation criteria of testicular tissues were seminiferous tubule degeneration, vacuolization, vascular congestion, and presence of immature cells shedding into the lumen. Testicular tissue sections prepared separately for all rats were evaluated blindly by two histopathologists by considering 10 different non-overlapping areas at × 10 magnification. A histological scoring table with a maximum of 12 points was established based on the existence of histological criteria (0 = none, 1 = less, 2 = moderate, and 3 = more) [26].

Additionally, testicular tissue sections were assessed using the Johnsen score (JS), which evaluates the loss of mature spermatogenic cell types in testicular damage, the advancement of spermatogenesis, and the degeneration of germinal epithelial cells. Briefly, in this scoring system, 40 seminiferous tubules randomly selected from testicular tissue sections were scored from 1 to 10 according to the severity of damage. Seminiferous tubules in which spermatogenic germ cells and Sertoli cells were absent were given a score of 1, while seminiferous tubules in which spermatogenic germ cells were present and spermatogenesis was completed with the normal histological structure were given a score of 10 (Table 1) [27].

**Table 1:** Johnsen's score used to evaluate testicular biopsies (JS)

*Score Description*
***1—****No cells*
***2—****Sertoli cells without germ cells*
***3—****Only spermatogonia*
***4—****Only a few spermatocytes*
***5—****Many spermatocytes*
***6—****Only a few early spermatids*
***7—****Many early spermatids without differentiation*
***8—****Few late spermatids*
***9—****Many late spermatids*
***10—****Full spermatogenesis*

### Immunohistochemical Evaluations

In testicular tissues, BcL2 (Sun Red, 201r.5304, China), Interleukin (IL)-1β (Santa Cruz, sc-1251, USA), TNF-α (Elabscience, BL3376, USA), Cysteine-aspartic protease 3 (Casp3) (Bioss, bs0081R, China), OTULIN (Boster, A07938-1, CA), NF-κB (Affinity, AF5006, USA), Dynamin related protein 1 (Drp1) (Bioss, bs-4100R (DLP1), USA), Mitofusin (Mfn) 2 (Bioss, bs-2988R-TR, USA) immunoreactivities were detected using the Avidin Biotin Peroxidase Complex method as described previously [28]. Mayer Haematoxylin staining was used for contrast staining of all sections. The prepared testicular tissue sections were examined and photographed under a light microscope (Leica, DM2500-MC170 HD, Germany). In the evaluation of immunoreactivities, immunoreactivities were calculated by considering the prevalence of immunostaining (*0 points were given for no immunostaining, 0.5 points for very mild immunostaining, 1 point for mild immunostaining, 2 points for moderate immunostaining, and 3 points for sections with high immunostaining severity*) X the severity of immunostaining (*0.1 for* < *25% immunostaining, 0.4 for 26–50% immunostaining, 0.6 for 51–75% immunostaining, 0.9 for 76–100% immunostaining*) [28].

### Terminal Deoxynucleotidyl Transferase Mediated Deoxyuridine-Biotin Nik End Labelling (TUNEL) Test Method

TUNEL is a DNA fragmentation detection technique that labels the 3′ hydroxyl terminal to detect double-stranded DNA breaks during apoptosis. Apoptotic cells were identified using the ApopTagPlus Peroxidase In Situ Apoptosis Detection Kit (Chemicon, Cat: S7101, USA). Blue nucleated cells in the TUNEL test were regarded as normal, healthy cells, whereas brown nucleated cells were regarded as TUNEL-positive apoptotic cells. In 25 randomly chosen fields, a total of 250 cells were counted. As mentioned in a prior study, the apoptotic index (%) was computed by considering the assessment results [26].

### Quantitative Real Time PCR

RNA isolation was performed according to the RNA isolation commercial kit protocol (High Pure RNA Tissue Kit Roche, Cat: 12,033,674,001). The purity and integrity of the obtained RNAs were checked by nanodrop device and gel electrophoresis. cDNA elution, Transcriptor First Strand cDNA Synthesis Kit (Roche Brand Cat: 04896866001) was used for cDNA Synthesis according to the commercial kits protocol. This mixture was kept in a Thermal Cycler at 25 °C for 10 min, 50 °C for 60 min, and 85 °C for 5 min to obtain cDNAs.

In the Real-Time PCR study, the primer sequences in Table 2 were used for GAPDH, reference gene and NF-κB, OTULIN, Casp3, and target genes. DNA Master SYBR Green I kit was used as the master mix in the real-time PCR study. PCR mixture protocol; ddH_2_O 12.4 µL, Mg + 2 (25 Mm) 1.6 µL, DNA Master SYBR Green I 10 × conc 2.0 µL, Primer Forward (Target or GAPDH) 1.0 µL, Primer Reverse (Target or GAPDH) 1.0 µL, total mixture volume 18 µL and adding 2.0 µL of each Target cDNA or GAPDH to this mixture, the final concentration volume was 20 µL. The Real-Time PCR protocol was 1 cycle at 95 °C for 30 s, 45 cycles at 95 °C for 0 s, 50 °C for 30 s, 72 °C for 20 s and 1 cycle at 40 °C with a 30 s cool. For each sample, 5 µl of cDNA was added to mixtures and run with Real-Time PCR (Roche-Lightcycler 96 USA).

**Table 2:** qRT-PCR primers

Symbol	Primer sequence (5’-3’)		Size (bp)		Accession no
GAPDH^a^	**F**	GACCCCTTCATTGACCTCAAC		137	NM_017008.4
	**R**	CGCTCCTGGAAGATGGTGATGGG			
NF-κB	**F**	GATCCTTTCGGAACTGGGCA		113	NM_001276711.2
	**R**	AGGTATGGGCCATCTGTTGAC			
OTULIN	**F**	CGCGAATGAGTCGGGGAAT		177	NM_001302889.1
	**R**	CACGGTACATGTCCTCCTCG			
CASP3	**F**	GGAGCTTGGAACGCGAAGAA		169	NM_012922.2
	**R**	ACACAAGCCCATTTCAGGGT			

Abbreviations: F, Forward primer; R, Reverse primer

^a^Used as reference gene for NF-κB, OTULIN and CASP3 gene expression levels

### Tissue Lysate Preparation and Western Blot Analysis

Testicular tissue was minced and lysed in a glass homogenizer using lysis buffer containing 50 mM Tris HCl, 150 mM NaCl, 1.0% (v/v) NP-40, 0.5% (w/v) Sodium Deoxycholate, 1.0 mM EDTA, 0.1% (w/v) SDS and 0.01% (w/v) sodium azide at a pH of 7.4. The samples were centrifuged at 15,000 rpm for 60 min, and the supernatant was collected and kept at -80° C. The total protein concentrations were measured by the Bradford method [29]. Protein samples (25 μg) were run on 12% sodium dodecyl sulfate–polyacrylamide gel electrophoresis (SDS-PAGE). After electrophoresis, proteins were transferred from the gel onto polyvinylidene difluoride membranes (PVDF, Millipore, Germany/USA; cat. No. IPVH00010). Membranes were blocked in a 5% non-fat milk powder, which was then incubated with primary antibodies (Anti-OTULIN, Boster, A07938-, 0.20 µg/ml / Anti-GAPDH, Santa Cruz – 1:3,000,, CA, USA) at 4° C/overnight. After incubation with the primary antibodies, the membranes were washed in TBST and then incubated with secondary antibodies (Anti-mouse IgG-HRP and Anti-rabbit IgG-HRP- Advansta 1:5,000). Subsequently, membranes were developed by enhanced chemiluminescence (ECL) and were visualized by X-Ray device [30].

### Statistical Analyses

Statistical analyses of the data collected for the study were carried out with the SPSS 22.0 package. The Shapiro–Wilk test was used to determine if the data was suitable for a normal distribution. For statistical examination of normally distributed data, the One-way ANOVA post hoc TUKEY test was employed. Kruskal–Wallis followed by Mann–Whitney U pairwise comparison tests were used for statistical analysis of non-normally distributed data. In quantitative Real-Time PCR analyses, results were expressed as fold increase and evaluated according to the 2(^ΔΔ^CT) method. A difference was considered statistically significant if it was *p* < 0.05. GraphPad Prism 8.4 was used to make a graphic presentation of the data collected during the investigation.

## Results

### Effects of CS and/or TQ Applications on Oxidant-Antioxidant Parameters in Testicular Tissue

**Fig. 1:**
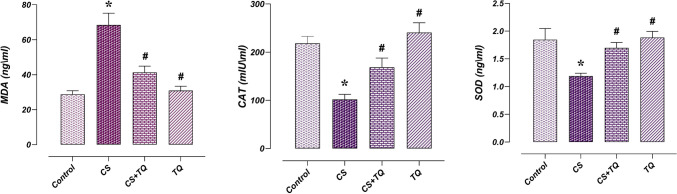
Effect of CS and\or TQ applications on oxidant/antioxidant parameters in testicular tissue Oxidant/antioxidant parameters in testicular tissues of control and TQ groups were similar. When the CS group was compared with the control group, an increase in MDA level and a decrease in CAT and SOD levels were observed. In the CS + TQ group, it was determined that the oxidant/antioxidant parameters that changed due to CS were regulated. *; compared to the control group (*p* < 0.05), #; Compared with the CS group (*p* < 0.05). CS; Cisplatin, TQ; Thymoquinone, MDA; Malondialdehyde, CAT; Catalase, SOD; Superoxide dismutase

There was no difference between testicular tissue MDA, CAT, and SOD levels in the control and TQ groups. In the CS group, MDA levels increased while CAT and SOD levels decreased compared to the control group (*p* < 0.05). However, in the CS + TQ group, MDA levels in testicular tissues decreased while CAT and SOD levels increased compared to the CS group (*p* < 0.05) (Fig. 1).

### Hormonal Effect of CS and/or TQ Applications

**Fig. 2:**
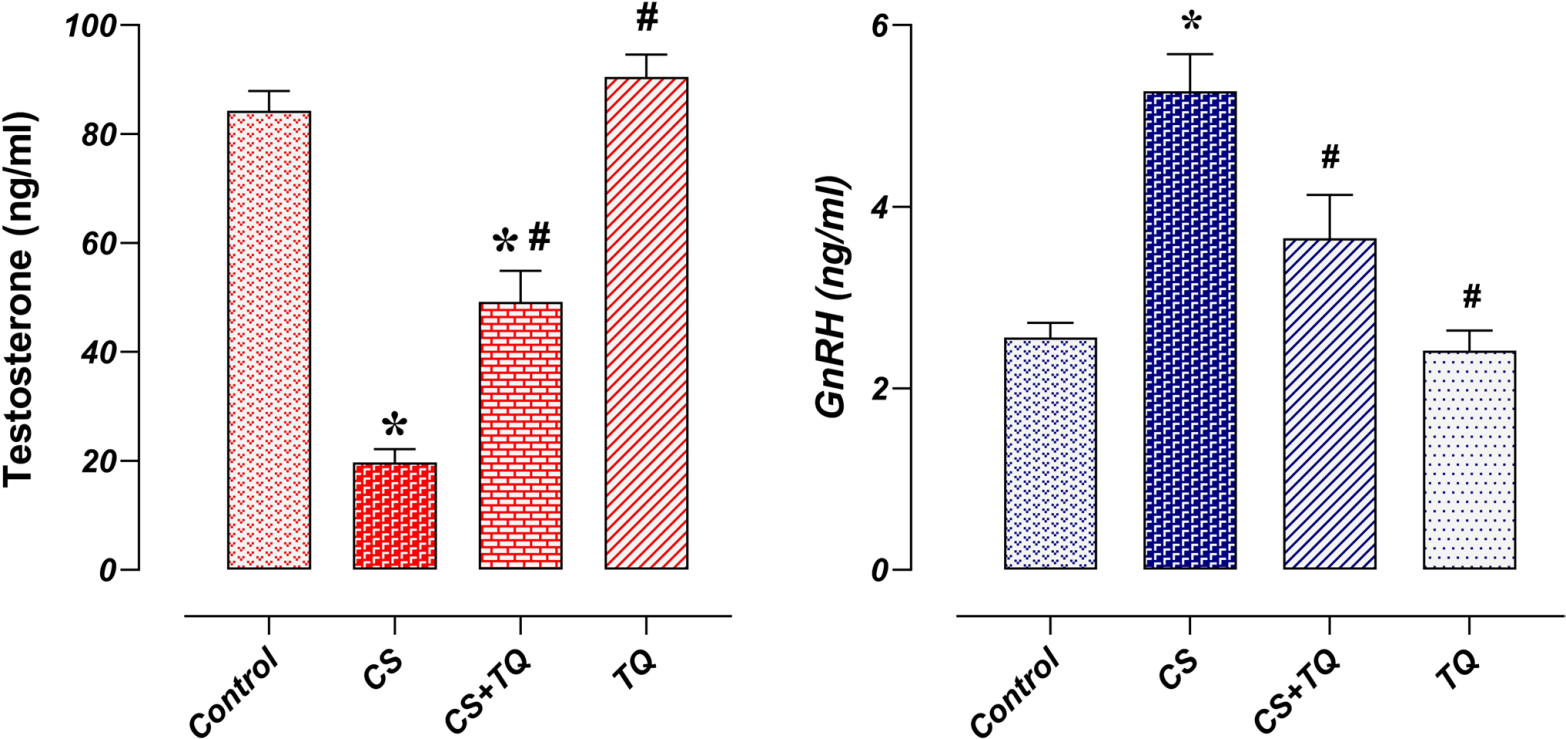
Effect of CS and\or TQ applications on reproductive hormones Serum testosterone and GnRH levels were similar in the control and TQ groups. GnRH levels increased while testosterone levels decreased in the CS group compared to the control group. In the CS + TQ group, it was determined that while testosterone levels increased compared to the CS group, GnRH levels decreased. *; compared to the control group (*p* < 0.05), #; Compared with the CS group (*p* < 0.05). CS; Cisplatin, TQ; Thymoquinone, GnRH; Gonadotropin-releasing hormone

There was no difference between testosterone and GnRH levels in the control and TQ groups. In the CS group, testosterone levels decreased and GnRH levels increased compared to the control group (*p* < 0.05). In the CS + TQ group, testosterone levels increased and GnRH levels decreased compared to the CS group (*p* < 0.05) (Fig. 2).

### Effects of CS and/or TQ Applications on Testicular Histopathology

In the study, the final body weight, testicular weight, and relative testicular weight of the rats in the control and TQ groups were similar. In the CS group, testicular weights and relative testicular weights were decreased compared to the control group. On the other hand, testicular weights and relative testicular weight increased in the CS + TQ group compared to the CS group (Table 3).

**Table 3:** Effect of CS and/or TQ applications on rat body weight and testicular weight

	Control	CS	CS + TQ	TQ	p*
Body weight (g)	210.14 ± 10.07	188.57 ± 9.86^a^	197.42 ± 10.26	206.14 ± 12.94^b^	< 0.01
Testicle weight (g)	1.96 ± 0.24	0.98 ± 0.14^a^	1.50 ± 0.24^a b^	1.86 ± 0.11^b^	< 0.01
Relative testicle weight	0.93 ± 0.13	0.52 ± 0.089^a^	0.76 ± 0.12^a b^	0.90 ± 0.06^b^	< 0.01

Data are presented as mean ± standard deviation

^a^; compared with the control group (*p* < 0.05),

^b^; compared with the CIS group (*p* < 0.05)

p* ANOVA

**Fig. 3:**
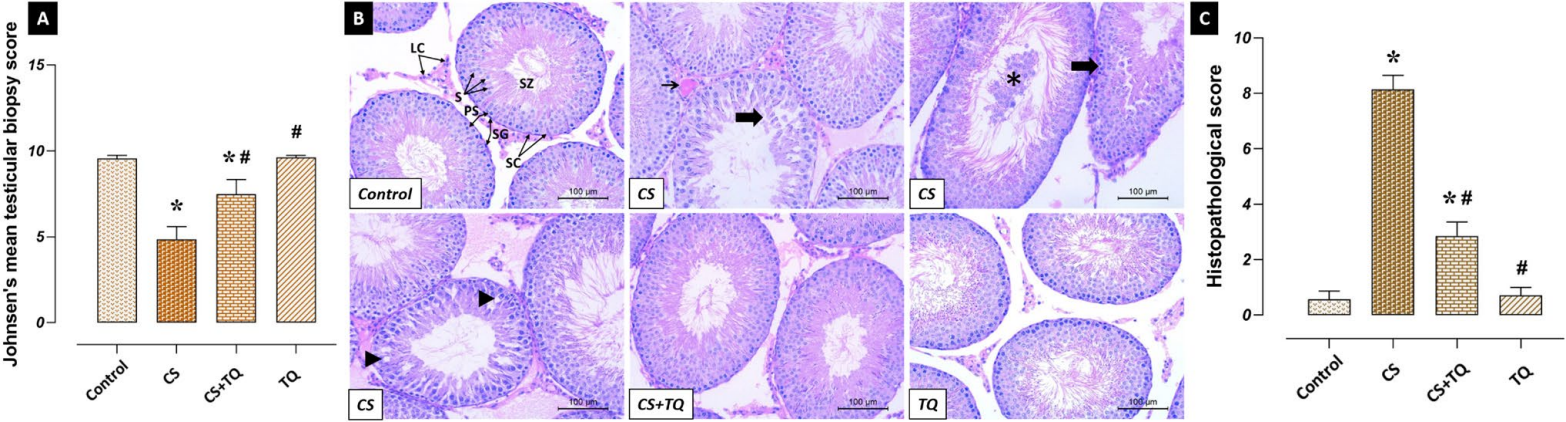
Histopathological effects of CS and/or TQ applications on testicular tissues: **A**; JS graph, **B**; histopathological microphotographs, **C**; histopathological score graph. Testicular tissues of the control and TQ groups had similar JS and normal histological structure. While JS and HS decreased in the CS group compared to the control group, histopathological damage increased. Thin arrow; vascular congestion, thick arrow; seminiferous tubule degeneration, triangle; vacuolization, star; immature cells shed into the lumen. In the CS + TQ group, it was determined that there was an increase in JS and a decrease in histopathological damage compared to the CS group. *; compared to the control group (*p* < 0.05), #; Compared to CS group (*p* < 0.05). Hematoxylin and eosin staining, scale bar; 100 μm. JS; Johnsen score, HS; histopathological score, CS; Cisplatin, TQ; Thymoquinone, LC; Leyding cells, SC; Sertoli cells, SG; Spermatogonia, PS; Primary spermatocytes, S; Spermatids, SZ; Spermatozoa

In the study, the normal histological structure was observed in testicular tissue sections of the control and TQ groups. Histopathological damages such as degeneration and vacuolization in the seminiferous tubules, vascular occlusion, and shedding of immature cells into the lumen were detected in the CS group compared to the control group (*p* < 0.05). However, it was determined that histopathological damage was reduced in the CS + TQ group compared to the CS group (*p* < 0.05). In addition, these results were confirmed by JS, another evaluation score of testicular tissue damage, which gave similar results (Fig. 3).

### Effects of CS and/or TQ Treatments On Mitochondrial Dynamics in Testicular Tissue

**Fig. 4:**
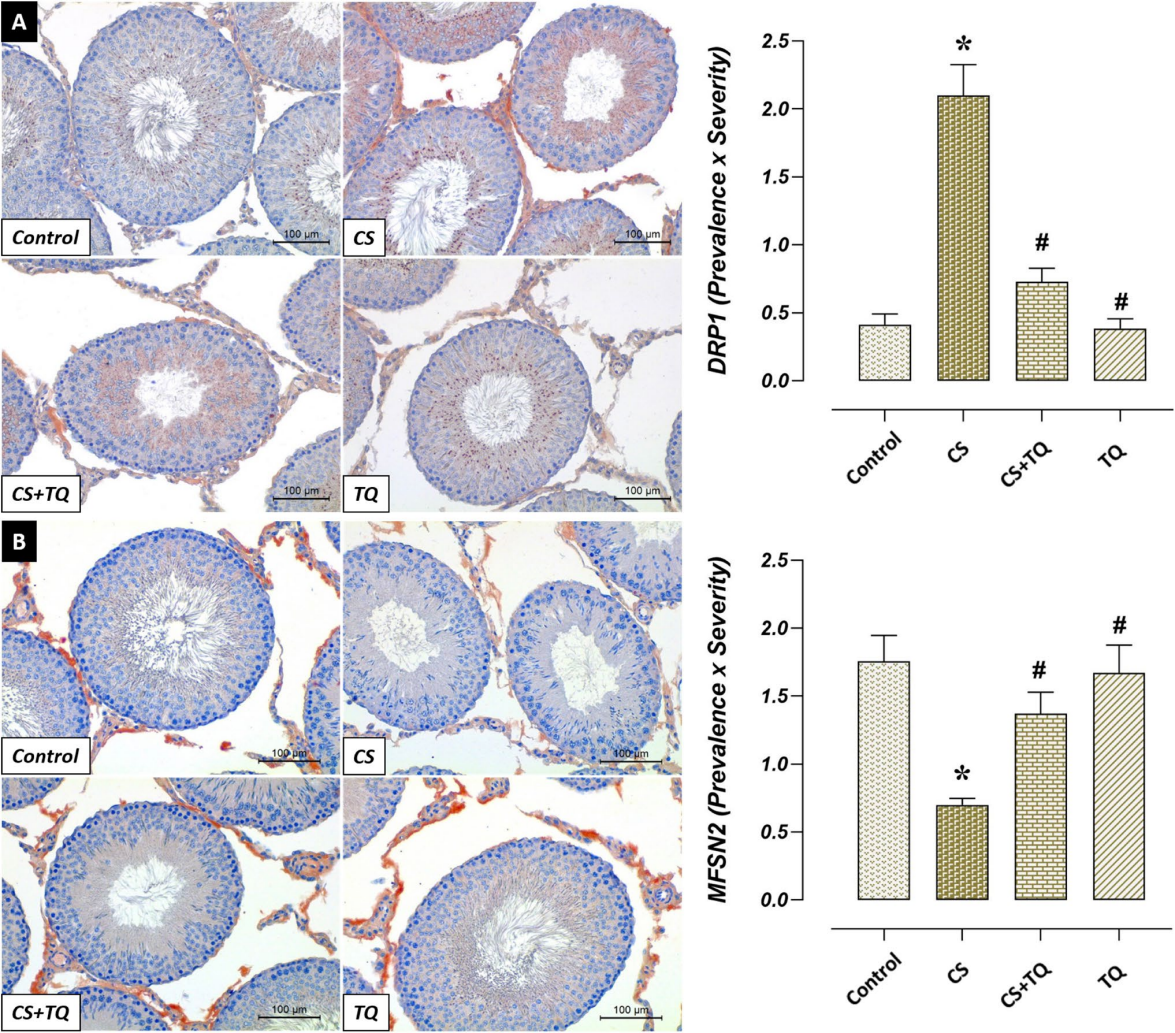
Effect of CS and/or TQ applications on immunoreactivity of mitochondrial dynamics in testicular tissues: **A**; Drp1 immunoreactivity microphotographs and graph, **B**; Mfsn2 immunoreactivity microphotographs and graph. Drp1 and Mfsn2 immunoreactivities in testicular tissues of control and TQ groups were similar. While Drp1 immunoreactivity increased in the CS group compared to the control group, Mfsn2 immunoreactivity decreased. In the CS + TQ group, Drp1 immunoreactivity decreased while Mfsn2 immunoreactivity increased compared to the CS group. *; compared to the control group (*p* < 0.05), #; Compared to CS group (*p* < 0.05). Drp1 and Mfsn2 immunohistochemical staining, scale bar; 100 μm. CS; Cisplatin, TQ; Thymoquinone, Drp1; Dynamin related protein 1, Mfsn2; Mitofusin 2

There was no difference between Drp1 and Mfn2 immunoreactivities of mitochondrial dynamics in testicular tissues of control and TQ groups. In the CS group, Drp1 immunoreactivity increased while Mfn2 immunoreactivity decreased compared to the control group (*p* < 0.05). However, Drp1 immunoreactivity decreased and Mfn2 immunoreactivity increased in the CS + TQ group compared to the CS group (*p* < 0.05) (Fig. 4).

### Effects of CS and/or TQ Treatments on İnflammatory Markers in Testicular Tissue

**Fig. 5:**
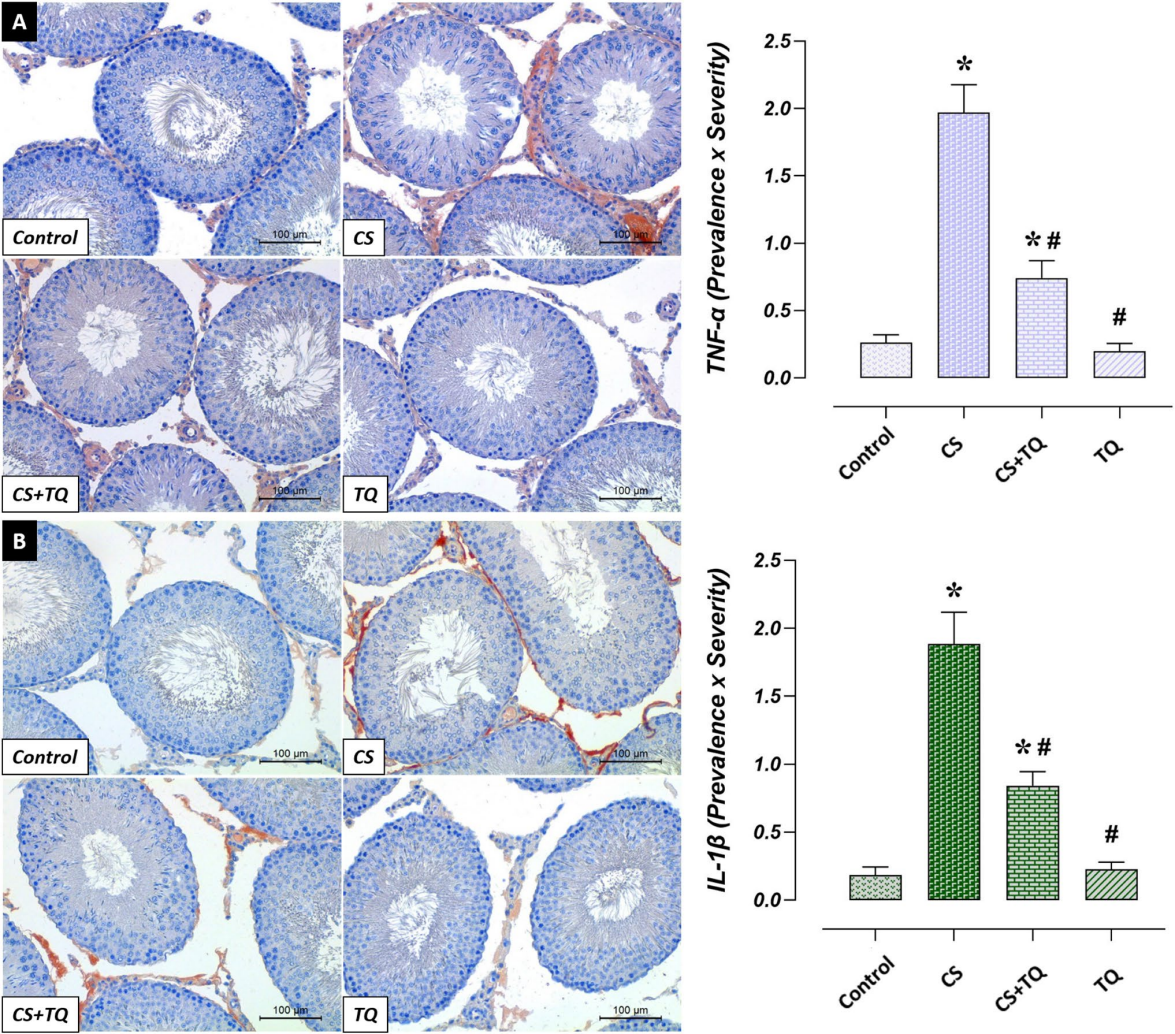
Effect of CS and/or TQ applications on inflammatory cytokine immunoreactivities in testicular tissues: **A**; TNF-α immunoreactivity microphotographs and graph, **B**; IL-1β immunoreactivity microphotographs and graph. TNF-α and IL-1β immunoreactivities in testicular tissues of control and TQ groups were similar. TNF-α and IL-1β immunoreactivities were increased in the CS group compared to the control group. In the CS + TQ group, the immunoreactivities of pro-inflammatory cytokines were decreased compared to the CS group. *; compared to the control group (*p* < 0.05), #; Compared to CS group (*p* < 0.05). TNF-α and IL-1β immunohistochemical staining, scale bar; 100 μm. CS; Cisplatin, TQ; Thymoquinone, TNF-α; Tumor necrosis factor-alpha, IL-1β; Interleukin 1 Beta

There was no difference between IL-1β and TNF-α levels in the testicular tissues of the control and TQ groups. IL-1β and TNF-α immunoreactivities were increased in testicular tissue in the CS group compared to the control group (*p* < 0.05). On the other hand, IL-1β and TNF-α immunoreactivities were decreased in testicular tissue in the CS + TQ group compared to the CS group (*p* < 0.05) (Fig. 5).

### Effects of CS and/or TQ Treatments on OTULIN Levels in Testicular Tissue

**Fig. 6:**
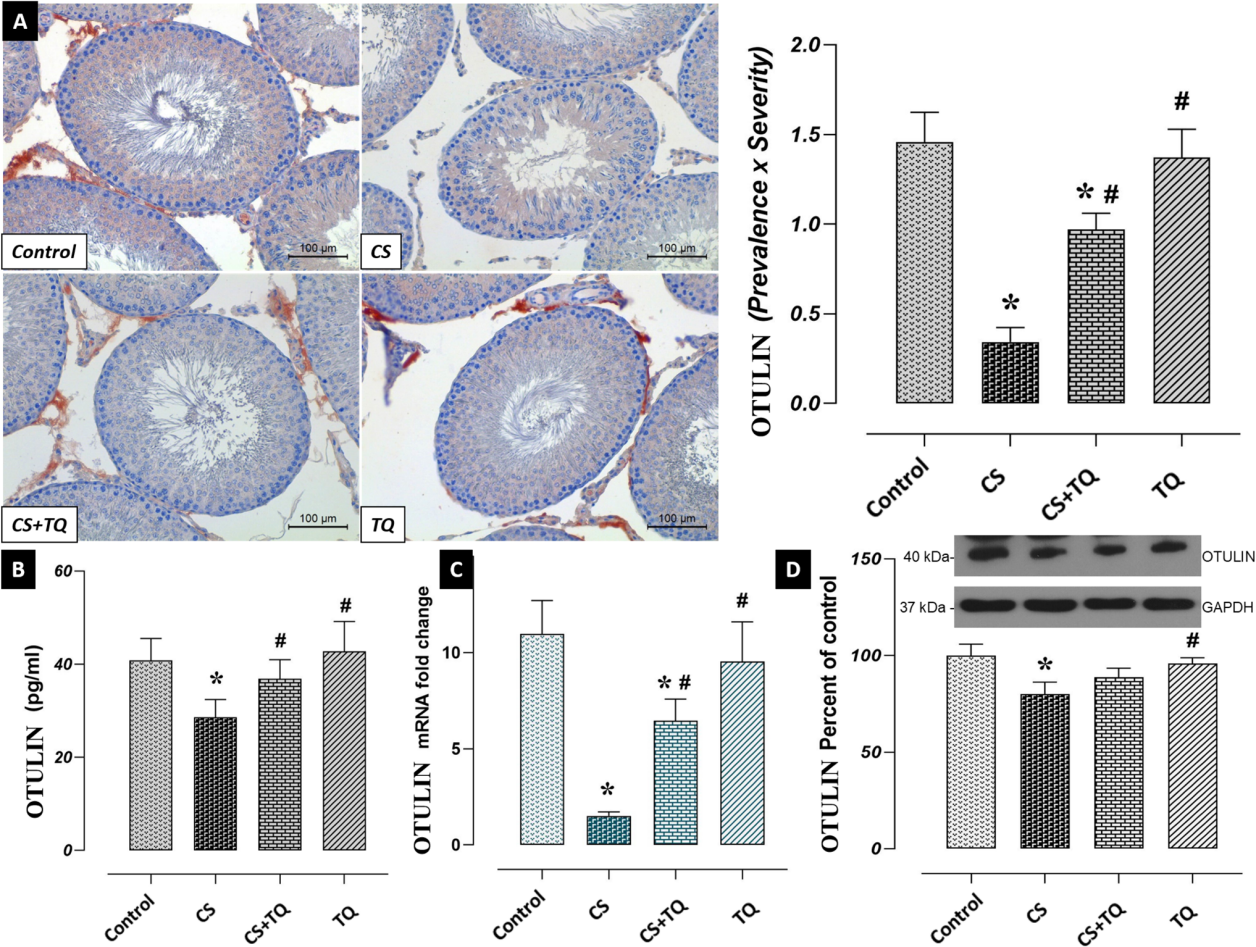
Effect of CS and/or TQ applications on OTULIN levels in testicular tissues: **A**; OTULIN immunoreactivity microphotographs and graph, **B**; OTULIN ELISA levels, **C**; OTULIN mRNA expression, **D**; OTULIN gene protein level. OTULIN levels in testicular tissues of control and TQ groups were similar. OTULIN immunoreactivity, ELISA level, mRNA expression and gene protein level were decreased in the CS group compared to the control group. An increase in OTULIN levels was detected in the CS + TQ group compared to the CS group. *; compared to the control group (*p* < 0.05), #; Compared to CS group (*p* < 0.05). A; OTULIN immunohistochemical staining, scale bar; 100 μm. CS; Cisplatin, TQ; Thymoquinone

There was no difference between OTULIN levels in the testicular tissues of the control and TQ groups. OTULIN immunoreactivity, expression, and protein levels were decreased in the CS group compared to the control group (*p* < 0.05). However, OTULIN immunoreactivity, expression, and protein levels increased in the CS + TQ group compared to the CS group (*p* < 0.05) (Fig. 6).

### Effects of CS and/or TQ Treatments on NF-κB Levels in Testicular Tissue

**Fig. 7:**
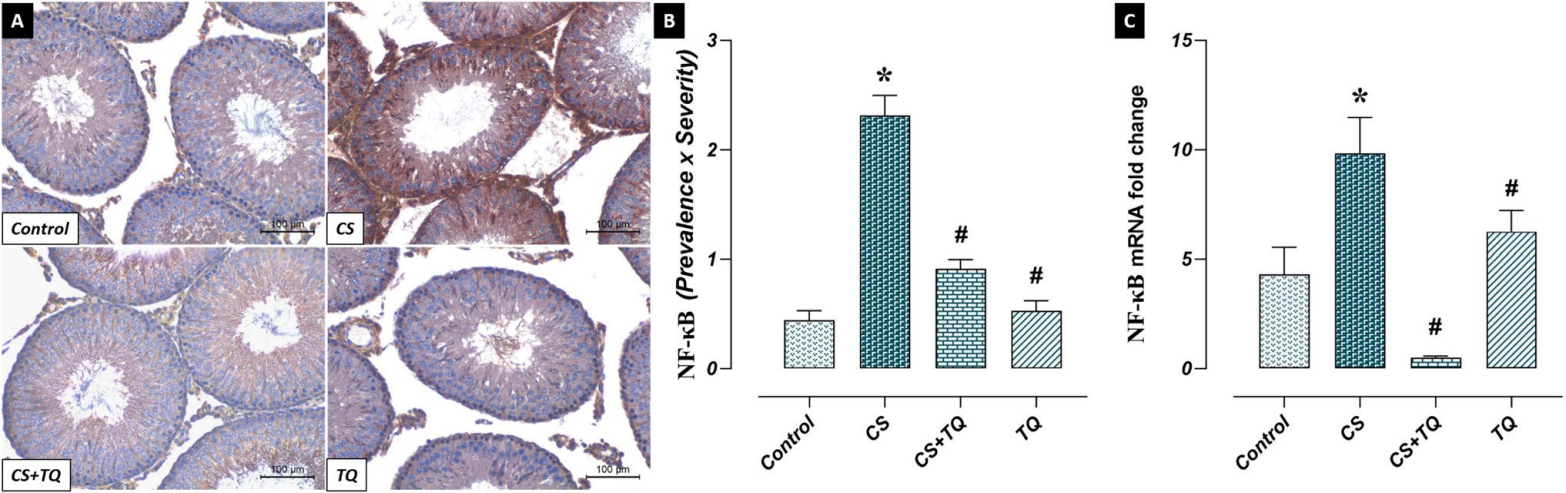
Effect of CS and/or TQ applications on NF-κB levels in testicular tissues: **A**; OTULIN immunoreactivity microphotographs, **B**; OTULIN immunoreactivity graph, **C**; NF-κB mRNA expression. NF-κB levels in testicular tissues of control and TQ groups were similar. NF-κB immunoreactivity and mRNA expression were increased in the CS group compared to the control group. A decrease in NF-κB levels was detected in the CS + TQ group compared to the CS group. *; compared to the control group (*p* < 0.05), #; Compared to CS group (*p* < 0.05). A; NF-κB immunohistochemical staining, scale bar; 100 μm. CS; Cisplatin, TQ; Thymoquinone

In the study, there was no difference between NF-κB levels in the testicular tissues of the control and TQ groups. NF-κB immunoreactivity and expression levels were increased in the CS group compared to the control group (*p* < 0.05). NF-κB immunoreactivity and expression levels decreased in the CS + TQ group compared to the CS group (*p* < 0.05) (Fig. 7).

### Effects of CS and/or TQ Applications on Apoptotic markers in Testicular Tissue

**Fig. 8:**
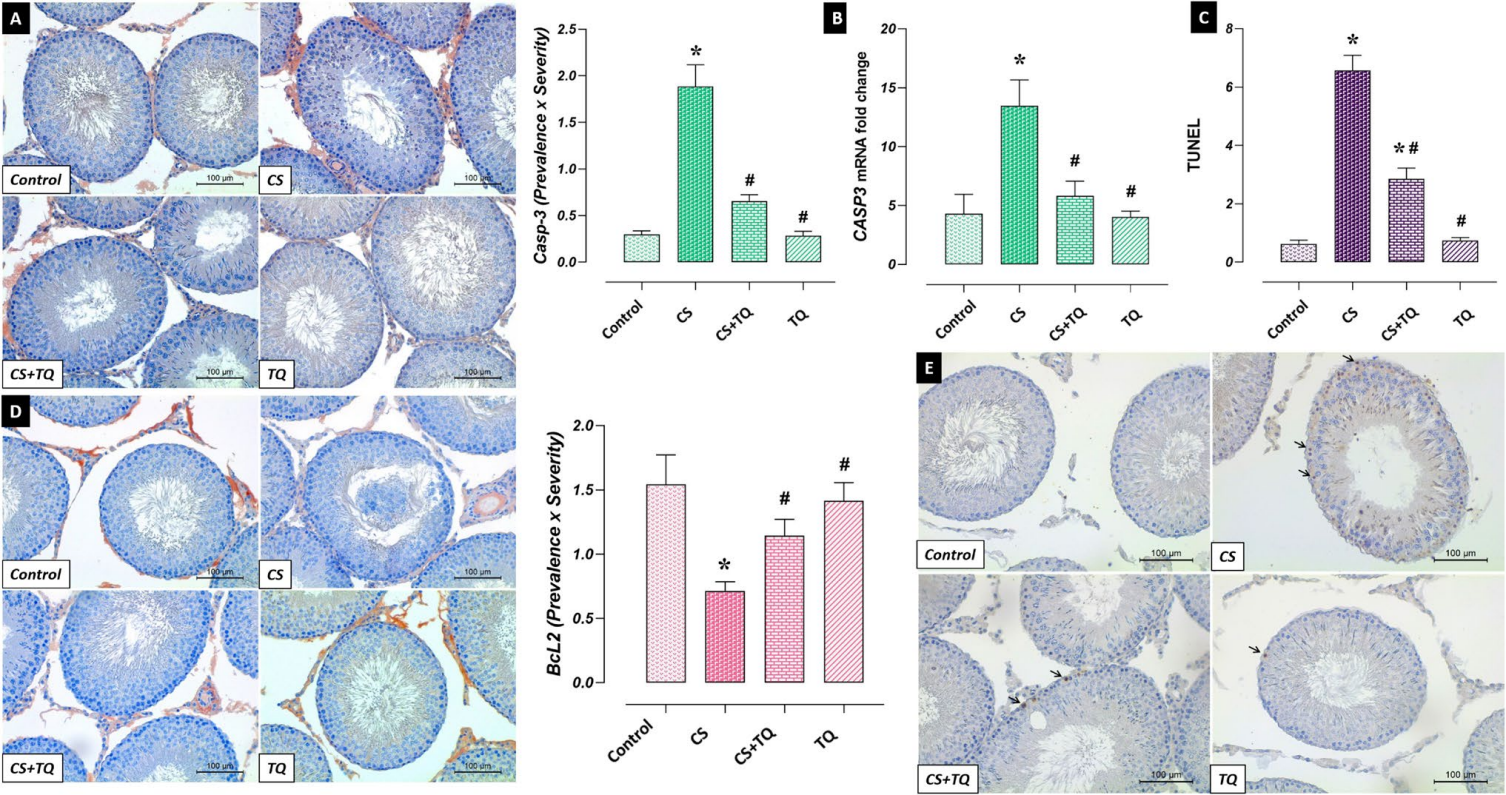
Effect of CS and/or TQ applications on apoptotic markers in testicular tissues: **A**; Casp3 immunoreactivity microphotographs and graph, **B**; Casp3 mRNA expression, **C**; TUNEL graph, **D**; BcL2 immunoreactivity microphotographs and graph, **E**; TUNEL microphotographs. Levels of apoptotic markers in testicular tissues of control and TQ groups were similar. It was determined that BcL2 immunoreactivity decreased in the CS group compared to the control group, while Casp3 levels and TUNEL-positive apoptotic cells increased. In the CS + TQ group, it was observed that BcL2 immunoreactivity increased compared to the CS group, while Casp3 levels and TUNEL-positive apoptotic cells decreased. *; compared to the control group (*p* < 0.05), #; Compared to CS group (*p* < 0.05). A; Casp3 immunohistochemical staining, D; BcL2 immunohistochemical staining, E; TUNEL staining, scale bar; 100 μm. CS; Cisplatin, TQ; Thymoquinone

Apoptotic Index (AI) calculated by considering anti-apoptotic BcL2, pro-apoptotic CASP3, and TUNEL-positive cells in testicular tissues of control and TQ groups were similar. In the CS group, BcL2 immunoreactivity decreased while AI, CASP3 immunoreactivity, and expression levels increased compared to the control group (*p* < 0.05). In contrast, the CS + TQ group showed an increase in BcL2 immunoreactivity and a decrease in AI, CASP3 immunoreactivity, and expression levels compared to the CS group (*p* < 0.05) (Fig. 8).

## Discussion

Although CS is a widely used antineoplastic agent in cancer treatment, it has many undesirable side effects, including testicular toxicity. Many times, these side effects lead to the termination of treatment or serious damage to other tissues and organs. In this context, many substances, especially herbal antioxidants, have been investigated and continue to be investigated against CS-induced toxic effects. However, a supplementary product that will eliminate or alleviate these undesirable effects has still not been found. In this context, TQ, which has proven to have many health-beneficial effects, especially antioxidant and anti-inflammatory, is promising. This is, as far as we know, the first study examining the effect of TQ on mitochondrial dynamics, TNF-α, OTULIN, and NF-κB levels against CS-induced testicular tissue damage. We report a therapeutic effect of TQ against CS-induced testicular tissue damage by regulating reproductive hormones, oxidative stress parameters, inflammation, mitochondrial dynamics, OTULIN, and suppressing apoptosis.

Although chemotherapy is an effective treatment method, it is considered a method with many toxic effects because it destroys cancer cells and damages healthy cells [31]. High dosages of chemotherapy medications are frequently utilized in clinical settings to treat a variety of tumor forms. Still, spermatogenic cells have been demonstrated to be harmed by chemotherapeutic medicines, even at low dosages [32]. By producing ROS, CS induces oxidative stress, which ultimately results in necrosis and cell death [3]. Studies have reported that CS significantly increases MDA levels, which are frequently used as an indicator of oxidative stress. However, it has also been reported in many studies that CS decreases SOD and CAT activities in testicular tissue [33, 34]. These findings supported the study's conclusion that CS treatment raised the MDA level in testicular tissue while lowering the levels of SOD and CAT. However, studies have shown that TQ suppresses oxidative stress caused by many drugs such as Paclitaxel [35], Cyclophosphamide [24] in testicular tissue. The reduced form of TQ (thymohydroquinone) acts as an electron donor to hydroxyl radicals (OH^−1^) and superoxide radicals that attack polyunsaturated fatty acids in the cell membrane. This explains the strong ROS scavenging capacity of TQ [36]. In addition, studies have reported that TQ is effective in reducing oxidative stress against CS-induced neuronal, hepatic, renal, and pulmonary toxic effects in rats [9, 37–39]. Similar to this, TQ supplementation in the current study reduced oxidative stress in testicular tissues caused by CS.

According to reports, testosterone levels decreased while oxidative stress increased in testicular damage due to CS administration [34, 40]. In this study, it was detected that testosterone levels decreased in CS-induced testicular tissue damage. However, Leydig cells are stimulated to produce testosterone in response to elevated GnRH levels. A negative feedback loop triggered by elevated testosterone levels inhibits GnRH production [41]. In the present study, it was detected that GnRH level increased in CS-treated rats. This may be associated with a decrease in testosterone levels as observed in our results. Furthermore, the rise in ROS that CS induces is responsible for the hypogonadism that it produces [42]. However, in the present study, it was detected that testosterone levels increased and GnRH levels decreased in rats administered TQ after CS. This may represent the antioxidant potential of TQ. In addition, the weight of the testicles is related to the number of Sertoli cells and sperm production. Testicular weight or size reflects the number of germinal cells in testicular tissues. In this study, it was shown that CS application reduced testicular weight. These results are consistent with previous studies [4, 17]. Testicular tissue damage causes severe reduction of cells involved in spermatogenesis, testosterone production, as well as Sertoli cells. Additionally, this results in impaired sperm production and disruption of the androgen-mediated negative feedback system of GnRH. This may cause an increase in LH and FSH levels [43]. In this study, TQ was also shown to increase testicular weight by reducing testicular tissue damage. This situation contributed to the regulation of TQ, CS-induced changing GnRH and androgen hormone levels.

In addition to CS-induced impaired steroidogenesis, oxidative damage in testicular tissue, and hypogonadism may explain the decrease in JS in rats [44]. Similarly, this study showed a decrease in JS and an increase in histopathological damage (HS) in CS-induced testicular damage. These results are consistent with studies reporting degeneration in the seminiferous tubule germinal epithelial layer, edema in the interstitial regions, vascular occlusion, as well as a decrease in the number of spermatids, spermatocytes and especially spermatozoa in CS-induced testicular tissue with a decrease in JS [33, 40, 44]. However, in this study, TQ supplementation in CS-induced testicular damage increased JS and reduced histopathological damage.

To maintain mitochondrial number, form, and function in physiological conditions, the cell's balance of mitochondrial dynamics (fusion and fission) is required [45]. Additionally, mitochondria provide energy for spermatogenesis. ROS are a byproduct of normal and unhealthy cellular functions that take place in the mitochondria [46]. Oxidative stress is exacerbated by aberrant conditions that disturb the balance of mitochondrial dynamics, leading to an increase in ROS. From mitochondrial dynamics, Drp1 is associated with mitochondrial fission, while Mfn2 is a protein associated with mitochondrial fusion [47]. In this current study, it was found that Drp1 immunoreactivity increased and Mfn2 immunoreactivity decreased in CS-induced testicular damage. Excessive mitochondrial fission and decreased fusion observed in this current study may be one of the effective factors in the development and exacerbation of oxidative stress, which is an important point in CS-induced testicular damage. This provides evidence for the idea that mitochondrial fission leads to an increase in mitochondrial ROS production, while mitochondrial fusion is linked to a decrease in ROS production [48]. However, in this current study, TQ supplementation in CS-induced testicular damage decreased Drp1 immunoreactivity and increased Mfn2 immunoreactivity. TQ's regulation of mitochondrial dynamics may be linked to its suppression of oxidative stress, as observed in our results.

Though the exact mechanism of CS toxicity is unknown, it is believed to originate from inflammation and ROS-mediated activation of cytokines, nuclear transcription factors, or caspase-dependent death [49]. According to a recent study, CS raised testicular pro-inflammatory cytokine levels, such as TNF-α and IL-1β [40]. Similarly, in this current study, an increase in CS-induced TNF-α and IL-1β levels was detected in testicular tissue. The development of signaling complex I is triggered by TNF's interaction with its receptor TNFR1 at the plasma membrane. This complex then activates the transcription factor NF-κB, which is subject to strict regulation through various phosphorylation and ubiquitination events [50]. It is well established that NFκB is essential for both the stress-induced adaptive response and the transcriptional pathway that produces cytokines [9]. As a marker of enhanced affinity between mitochondria and the nucleus and mitochondrial NF-κB activation, LUBAC-mediated M1-ubiquitination takes place [51]. It has been reported that OTULIN regulates mitochondrial M1 proliferation [52]. However, OTULIN deficiency inhibits LUBAC activity, leading to dysregulation of TNF-induced complex I. This triggers the formation of complex II, resulting in increased cell death [20, 23]. In this current study, it was detected that OTULIN levels decreased while NF-κB levels increased in CS-induced testicular tissue damage. The increase in CS-induced proinflammatory cytokines seems to be one of the reasons for this situation. However, a study reported that TQ suppressed NFκB in inflammatory microglia cells [53]. It has been reported that TQ reduces TNF-α and NFκB expressions in a rat arthritis model [54]. Another study reported that TQ significantly reduced TNF-α and NF-κB transcription in the testes of hypothyroid rats [14]. Similarly, in this current study, it was determined that TQ reduced the increased TNF-α and NF-κB levels caused by CS in the testicular tissue, and also increased the decreased OTULIN level.

In a study, the inhibitory effect of Cilostazol on Toll-like receptor (TLR) signal-mediated NF-κB activation was attributed to its anti-inflammatory effect [55]. Similarly, in the current investigation, the regulatory effect of TQ on OTULIN and NF-κB levels may be related to its anti-inflammatory effect. As shown in our study, the decrease in pro-inflammatory (TNF-α and IL-1β) cytokine levels in CS-derived testicular tissue with TQ treatment strengthens this idea. However, NF-κB is thought to be a critical link between oxidative stress, inflammation, and apoptosis. Oxidative stress, together with increased expression of inflammatory mediators, has been shown to cause cell death in the testicles of animals exposed to CS through necrosis or apoptosis [56]. The imbalance in mitochondrial redox processes caused by CS leads to caspases-associated cell death [57]. Translocation of cytosolic BcL2-associated together, with these events triggers cell apoptosis when exposed to CS [58]. In this current study, anti-apoptotic BcL2 decreased while pro-apoptotic Casp3 levels increased in CS-induced testicular damage. In addition, in this current study, it was determined that TUNEL-positive apoptotic cells increased in testicular tissues due to CS application by the TUNEL method, which allows the determination of apoptotic cells. These findings aligned with a prior investigation that demonstrated notable rises in apoptosis-associated proteins, such as Casp3, in testicular tissues following CS treatment [41, 44]. Additionally, the same study reported that oxidative stress and TNF-α/NF-κB/Casp3 pathway activation increased in CS-induced testicular damage [59]. Similarly, in this current study, it was detected that oxidative stress parameters and the TNF-α/OTULIN/NF-κB pathway were negatively affected in CS-induced testicular damage. However, TQ treatment was effective in regulating CS-induced negativities in testicular tissues. Although the present study reveals important results, it has some limitations. The first of these is how TQ administration affects the antitumoural activity of CS while reducing CS-induced testicular tissue damage. Studies on this subject suggest that TQ supports the antitumoural effects of CS and similar chemotherapeutic drugs. For example, it has been reported that the combination of TQ and Gemcitabine in breast cancer cells significantly increased cancer cell apoptosis (%80.9) compared to gemcitabine alone (%22.7) [60]. Another study showed that TQ significantly enhanced the cytotoxicity of CS in 5637 human bladder cancer cells and reported that the TQ-CS combination might be a good alternative for bladder cancer treatment [61]. A different study reported that TQ potentiated the anti-cancer effects of CS in oral squamous cell carcinoma [62]. Another limitation of the present study is that spermiogram analysis was not performed.

As a result, CS caused oxidative stress, histopathological damage, inflammation, destabilization of mitochondrial dynamics, and an increase in apoptotic cells in testicular tissues, along with a decrease in testosterone levels. However, in testicular damage caused by CS, TQ supplementation demonstrated anti-oxidant, anti-inflammatory, and anti-apoptotic effects. In addition, TQ contributed to the alleviation of CS-induced toxic effects by regulating the TNF-α/OTULIN/NF-κB pathway. These important findings reveal the therapeutic potential of TQ to reduce the toxic effects of chemotherapeutic drugs. However, detailed cellular mechanisms need to continue to be investigated to fully understand the supportive and therapeutic aspects of TQ in CS-induced toxicities.

## Data Availability

Data will be provided upon reasonable request.
